# The Discovery of Novel PGK1 Activators as Apoptotic Inhibiting and Neuroprotective Agents

**DOI:** 10.3389/fphar.2022.877706

**Published:** 2022-03-21

**Authors:** Shao-Jia Qiang, Yu-Qi Shi, Tong-Yu Wu, Jing-Quan Wang, Xue-Lian Chen, Jie Su, Xin-Ping Chen, Jia-Zhong Li, Zhe-Sheng Chen

**Affiliations:** ^1^ School of Pharmacy, Lanzhou University, Lanzhou, China; ^2^ Department of Pharmaceutical Sciences, College of Pharmacy and Health Sciences, St. John’s University, Queens, NY, United States

**Keywords:** stroke, neuroprotective, PGK1, virtual screening, apoptosis

## Abstract

Stroke is the second leading cause of death worldwide and the leading cause of long-term disability that seriously endangers health and quality of human life. Tissue-type fibrinogen activator is currently the only drug approved by FDA for the treatment of ischemic stroke. Neuroprotection is theoretically a common strategy for the treatment of both ischemic and hemorrhagic stroke; therefore, the development of neuroprotective agent has been the focus of research. However, no ideal neuroprotective drug is clinically available. Phosphoglycerate kinase-1 (PGK1) activator has the effect of inhibiting apoptosis and protecting tissue damage, and therefore could be a potential neuroprotective agent. To obtain effective PGK1 activators, we virtually screened a large chemical database and their evaluated the efficacy by the *Drosophila* oxidative stress model, PGK1 enzymatic activity assay, and oxygen-glucose stripping reperfusion (OGD/R) model. The results showed that compounds 7979989, Z112553128 and AK-693/21087020 are potential PGK1 activators with protective effects against PQ-induced oxidative stress in the *Drosophila* model and could effectively ameliorate apoptosis induced by OGD/R-induced neuronal cell injury. Additionally, compounds 7979989 and Z112553128 are effective in alleviating LPS-induced cellular inflammation. This study indicated that these compounds are promising lead compounds that provide theoretical and material basis to the neuroprotective drug discovery.

## 1 Introduction

Stroke, one of the most prevalent and devastating diseases affecting human being all over the world, is the second leading cause of death and the leading cause of long-term disability worldwide (Liu et al., 2011; GBD 2016 Neurology Collaborators, 2019). Stroke is divided into ischemic and hemorrhagic strokes according to pathogenesis, of which 85% are ischemic strokes (Green and Shuaib, 2006). Acute ischemic stroke is a severe and life-threatening disease that most commonly involves acute neuronal cell death and delayed neuronal cell death caused by occlusion of large blood vessels. The latter is a form of programmed cell death, also known as apoptosis (Nitatori et al., 1995), which is the main pathological change following ischemic and hypoxic injury to brain tissue (Beilharz et al., 1995; Graham and Chen, 2001).

Apoptosis is the genetically controlled, autonomous, and orderly death of cells that maintain the stability of the internal environment. However, inappropriate apoptosis may be directly or indirectly associated with the development of a variety of diseases, including stroke (Graeber and Moran, 2002; Elmore, 2007; Yuan, 2009). If the development of neuro-apoptosis can be arrested, it is possible to reduce the extent of cerebral ischemia-reperfusion injury and reduce the extent of infarction. Current stroke therapeutic agents, including thrombolytics, anticoagulants, antiplatelet agents, and free radical scavengers, are designed to open occluded vessels and restore blood perfusion as soon as possible to save more tissue in the ischemic hemisphere and thereby improve neurological deficits (Herpich and Rincon, 2020; Ospel et al., 2020). Unfortunately, in reality, the effectiveness of the available therapeutic agents and tools are not promising (Liu et al., 2011; Green and Shuaib, 2006). Stroke scholars agree that a treatment model of revascularization combined with neuroprotection is the best strategy for treating stroke. However, there are no ideal neuroprotective drugs available clinically and many neuroprotective agents have failed to translate in clinical trials, making the development of stroke therapeutics a worldwide challenge (Yuan, 2009; Chamorro et al., 2016). Therefore, there is an urgent need to develop novel anti-apoptotic drugs with neuroprotective effects for stroke therapy.

The clinical drug terazosin has been reported to translate into the treatment of devastating diseases including stroke. Terazosin activates HSP90 *via* phosphoglycerate kinase-1 (PGK1), thereby enhancing tolerance to cellular stress, inhibiting apoptosis, and protecting stressed cells while alleviating stroke symptoms in rats (Chen et al., 2015). However, terazosin is used clinically as a hypotensive drug, and it can bring about the side effect of lower blood pressure while inhibiting apoptosis. Therefore, the activation of PGK1 by terazosin has some drawbacks. Although current studies have confirmed that PGK1 is a novel drug target against apoptosis and PGK1 activators has the potential to inhibit apoptosis and protect tissues from damage (Mazzoni et al., 2009; Han et al., 2010), but no better PGK1 activators have yet been identified. Therefore, if an ideal PGK1 activator can be identified and screened for apoptosis inhibitors, it will certainly be possible to develop neuroprotective drugs for the treatment of stroke.

In this study, we firstly used virtual screening technique to search for potential apoptosis inhibitors targeting PGK1 by screening Specs natural compound library and PubChem database. Then PQ-induced *Drosophila* oxidative stress model, PGK1 enzyme activity assay, and oxygen glucose deprivation reperfusion model in rat pheochromocytoma cells (PC12 cells) were used to evaluate the anti-oxidative, anti-inflammatory, and protective effects against *in vitro* simulated brain hemorrhage of the hit compounds. A lipopolysaccharide (LPS)-induced inflammation model in RAW264.7 cells was used to validate their anti-apoptotic effects.

## 2 Results

### 2.1 Virtual Screening of Apoptotic Agents

**FIGURE 1:**
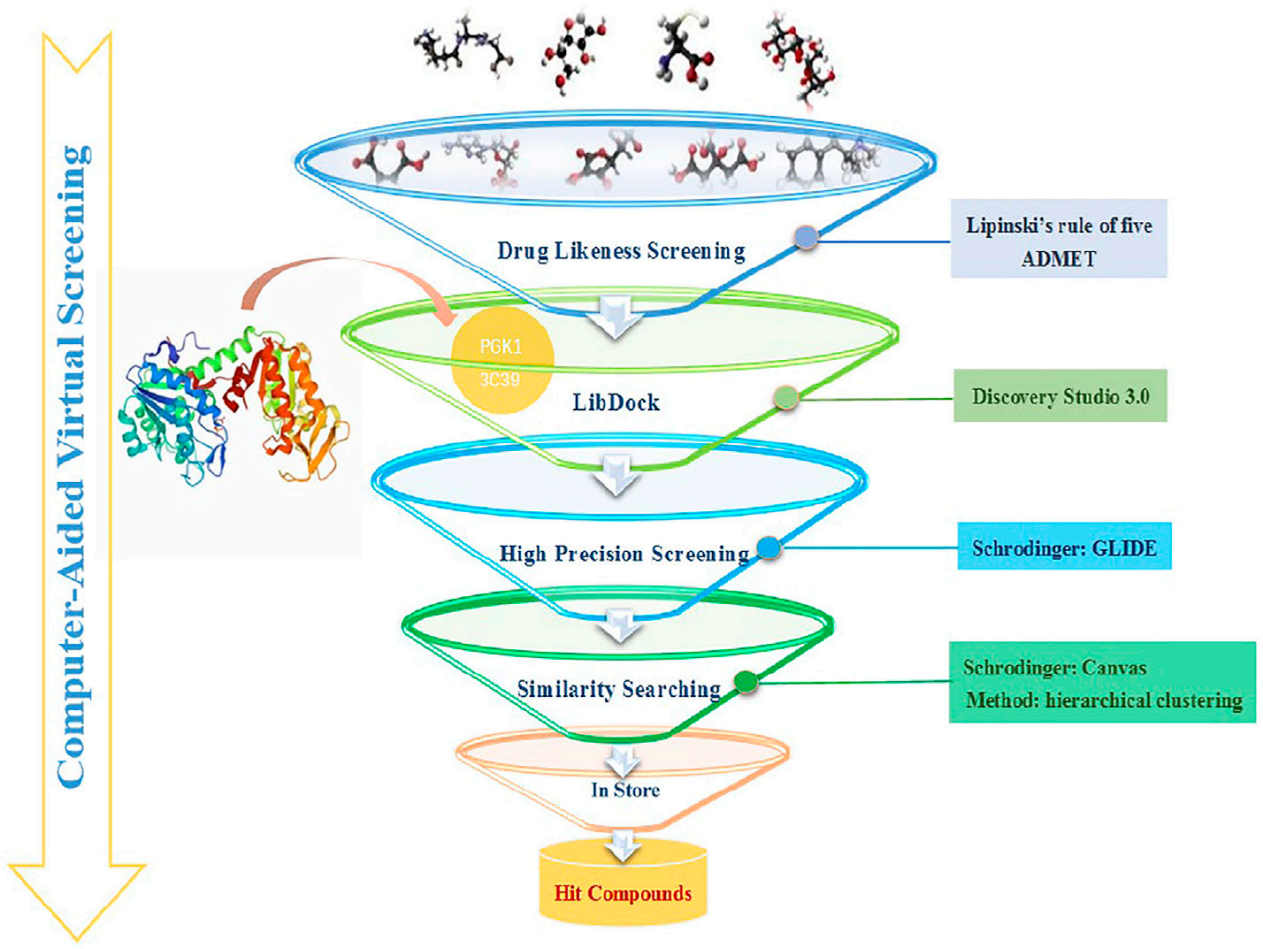
Virtual screening flow chart.

The combination of computational and experimental strategies is of great value for the identification and development of desirable compounds. Structure-based virtual screening (SBVS) has been used for decades in early drug discovery to search chemical compound libraries for novel bioactive molecules against a particular drug target (Kontoyianni, 2017). The computational technique underlying virtual screening, molecular docking, is widely used in modern drug design to explore ligand conformations at the binding site of macromolecular targets (Ferreira et al., 2015). The ligand-receptor binding free energy, estimated by assessing the ligand binding mode during intermolecular recognition and the corresponding intermolecular interactions, provides a scoring ranking of docked compounds based on the binding affinity of the ligand-receptor complex (Huang and Zou, 2010; Lopez-Vallejo et al., 2011). The technique is fast enough to allow virtual screening of ligand libraries containing tens of thousands of compounds in a short period of time (Forli et al., 2016). In this study, a virtual screening procedure was established as shown in Figure 1. Initially, 73355 compounds were filtered based on Lipinski’s rule of five and ADMET property prediction, and 35414 compounds remained. Next, molecular docking was performed using the Libdock module to perform a rough screening of the compounds and the top 4% compounds with the highest docking scores were retained. Then, an exact docking was performed using the Glide XP module and retained the top 20% compounds with the highest scores. Finally, hierarchical clustering was performed using the Canvas module to yield 20 groups of compounds, from where 19 compounds were selected by analyzing the receptor-ligand binding mode, ligand spatial site resistance and backbone characteristics of the compounds. We successfully purchased 11 of them (No 1-11 as listed in Table 1) for subsequent bioassays.

**TABLE 1:** Potential hit compounds obtain by virtual screening.

Number	ID	Name	Structure	Docking score
1	D510-0971	(4S)-4-[1-[(2R)-3-(2-chlorophenoxy)-2-hydroxypropyl]benzimidazol-2-yl]-1-(3-methoxyphenyl)pyrrolidin-2-one	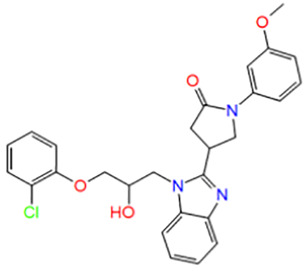	−5.403
2	9015355	3-(1,3-benzodioxol-5-yl)-5-[(4-chloro-2-methylphenoxy)methyl]-1,2,4-oxadiazole	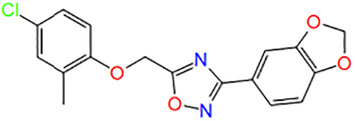	−5.002
3	7979989	N-[(1R)-1-[5-[2-(4-bromoanilino)-2-oxoethyl]sulfanyl-4-ethyl-1,2,4-triazol-3-yl]ethyl]-4-fluorobenzamide	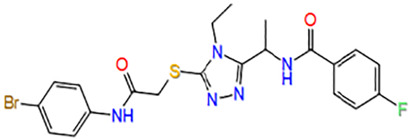	−5.273
4	STK513348	4-[[[4-[(2,4-dichlorophenyl)methoxy]-3-ethoxyphenyl]methylazaniumyl]methyl] benzoate	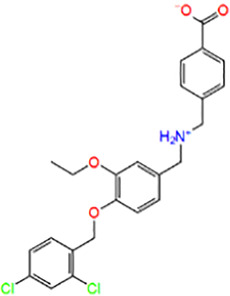	−8.086
5	Z112553128	(2R)-N-(2,3-dihydro-1H-inden-5-yl)-2-[[5-[(3-methylphenoxy)methyl]-4-prop-2-enyl-1,2,4-triazol-3-yl]sulfanyl] opanamide	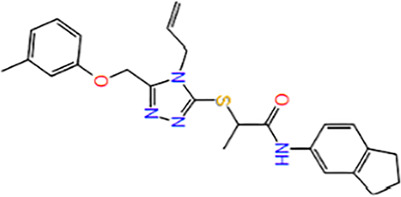	−4.578
6	PB169590210	N-[4-(3,4-difluorophenyl)-1,3-thiazol-2-yl]-3-(8-methyl-4-oxoquinazolin-3-yl)propanamide	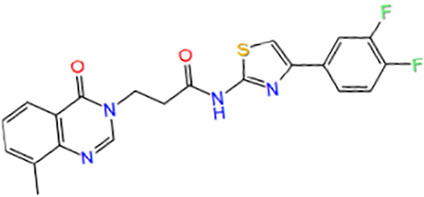	−4.333
7	F5326-0143	N-[4-[3-(4-benzylpiperazin-1-yl)-3-oxopropyl]-1,3-thiazol-2-yl]-4-fluorobenzenesulfonamide	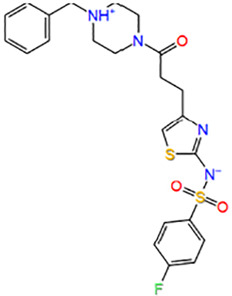	−5.617
8	F3260-0657	3,7-dimethyl-1-[(4-methylphenyl)methyl]-8-phenacylsulfanylpurine-2,6-dione	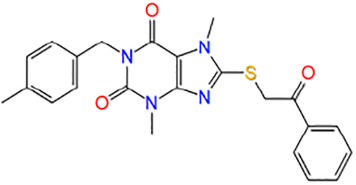	−4.442
9	AO-774/41465495	3-(3,4,5-trimethoxybenzyl)quinoxalin-2(1H)-one	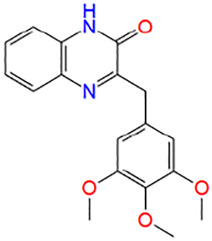	−5.552
10	AI-372/20970054	1-(2,4-dihydroxyphenyl)-2-phenylethanone	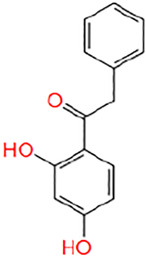	−4.642
11	AK-693/21087020	Clausenidin	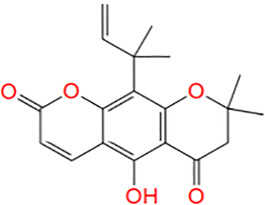	−4.395
12	AL-466/21162052	Smilagenin (Isosarsapogenin)	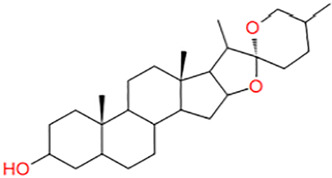	−4.339
13	ZINC16490691	N-[2-(3,3-diphenylpropylamino)-2-oxoethyl]-1,3-benzodioxole-5-carboxamide	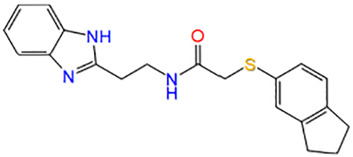	−5.957
14	ZINC16265293	N-[(4-fluorophenyl)methyl]-5-[2-(4-methoxyphenoxy)ethylsulfanyl]-1,3,4-thiadiazol-2-amine	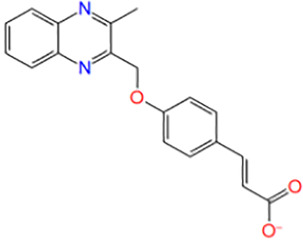	−5.145
15	ZINC16576908	Methyl-(2R,3R)-3-[3,4 bis(phenylmethoxy)phenyl]-2,3 dihydroxypropanoate	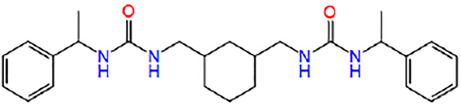	−4.468
16	ZINC16496026	(5-bromo-2-hydroxyphenyl)-[4-(2,3-dichlorophenyl)sulfonylpiperazin-1-yl]methanone	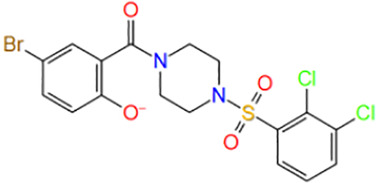	−6.451
17	ZINC16697027	1-[(1S)-1-phenylethyl]-3-[[(1R,3S)-3-[[[(1R)-1-phenylethyl]carbamoylamino]methyl]cyclohexyl]methyl]urea	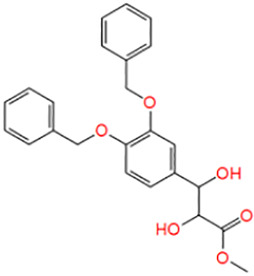	−5.331
18	ZINC16335082	(E)-3-[4-[(3-methylquinoxalin-2-yl)methoxy]phenyl]prop-2-enoic acid	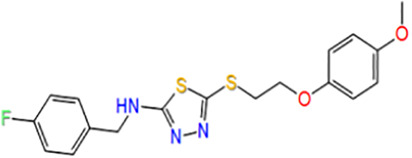	−7.594
19	ZINC16640765	N-[2-(1H-benzimidazol-2-yl)ethyl]-2-(2,3-dihydro-1H-inden-5-ylsulfanyl)acetamide	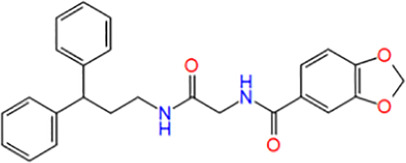	−6.758

### 2.2 Induction of Oxidative Stress Model

**FIGURE 2:**
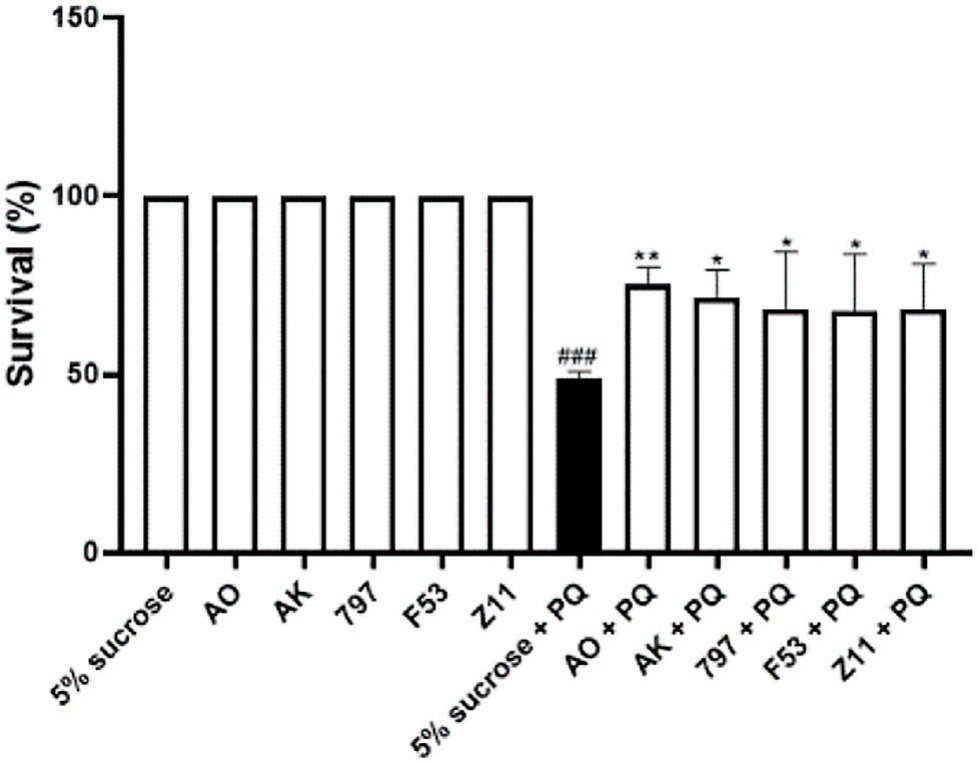
Identification of neuroprotective compounds in the PQ-induced oxidation model. 5% sucrose + 6 mg/ml PQ vs. 5% sucrose, ###*p* < 0.001, PQ at this concentration can have a lethal effect on *Drosophila*.; AO+ 6 mg/ml PQ vs. 5% sucrose + 6 mg/ml PQ, **p < 0.01; AK + 6 mg/ml PQ vs. 5% sucrose + 6 mg/ml PQ, *p < 0.05; 797 + 6 mg/ml PQ vs. 5% sucrose + 6 mg/ml PQ, *p < 0.05; F53 + 6 mg/ml PQ vs. 5% sucrose + 6 mg/ml PQ, *p < 0.05; Z11 + 6 mg/ml PQ vs. 5% sucrose + 6 mg/ml PQ, *p < 0.05; This result demonstrated that these five compounds had antioxidant effects and were effective in the PQ-induced oxidation model. Each bar represents the mean ± standard deviation (SD) calculated from three independent experiments.

We chose*Drosophila* as a screening platform, which is less expensive to raise, breed and screen than traditional mammalian models and has a shorter testing cycle (Pandey and Nichols, 2011). Studies have shown that the herbicide paraquat has neurotoxic effects (Chaudhuri et al., 2007) and *Drosophila* models exposed to PQ are often used as the models of neurodegenerative diseases to find new neuroprotective compounds (Jaiswal et al., 2012; Fernández-Hernández et al., 2016; Hajji et al., 2019). Here, *Drosophila* were treated with 6 mg/ml Paraquat dichloride (methyl viologen; PQ) in a sucrose solution, which provides basic energy for survival, for 24 h (Reiszadeh Jahromi et al., 2021). The data showed that 6 mg/ml PQ exposure led to lethal effects in *Drosophila*, and the survival rate was 50%. The survival rates of all groups with single addition of compounds were 100%, demonstrating that all screened compounds had no effect on the normal growth of *Drosophila* at this concentration. Compared with the experimental group (5% sucrose + 6 mg/ml PQ), the survival rate of *Drosophila* in the experimental group (compound + 6 mg/ml PQ) was improved. Five compounds showed significant protective effects against PQ-induced toxicity after pretreatment (Figure 2). Overall, these five compounds appear to be a potential neuroprotective candidate against PQ toxicity in PQ-induced toxicity model in *Drosophila*.

### 2.3 Compounds Increased PGK1 Kinase Activity

**FIGURE 3:**
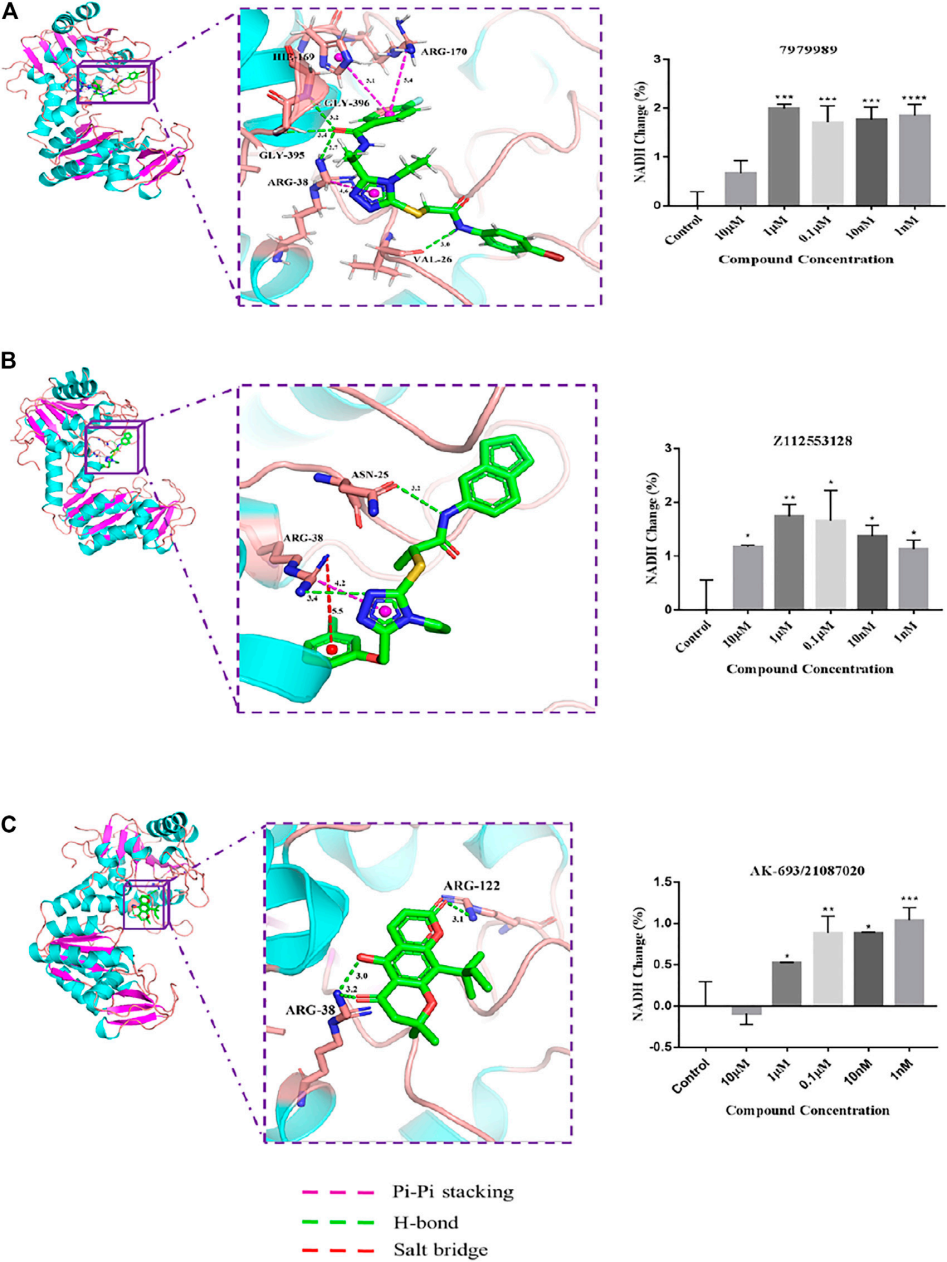
The intermolecular interaction of the predicted binding modes of **(A)** 7979989; **(B)** Z112553128; **(C)** AK-693/21087020. Effect of these three compounds on the activity of purified human PGK1 enzyme. ****p < 0.0001; ***p < 0.005, **p < 0.01, *p < 0.05 vs. control group. Each bar represents the mean ± standard deviation (SD) calculated from three independent experiments.

Experiments were designed to test the effect of compounds on the PGK-catalyzed reaction *in vitro* based on PGK kinase activity. As the PGK self-catalyzed reaction is rather rapid and not easily detectable, an indirect approach was used by coupling the PGK-catalyzed reaction with the pyruvate dehydrogenase (GAPDH) catalyzing the production of 1,3-BPG from glyceraldehyde phosphate. The substrate for the PGK forward reaction is 1,3-BPG, and if the PGK-catalyzed reaction is positively enhanced, which can cause a significant decrease in the concentration of 1,3-BPG in the reaction system, promoting the GAPDH forward reaction. The product of the GAPDH forward reaction, NADH, has strong absorption at 339 nm, and thus the strength of the PGK reaction can be monitored by the changes in NADH. To test the effect of compounds on the activity of PGK1, an *in vitro* assay was carried out. Purified hPGK1 was provided with substrates (ADP and 1,3-BPG) and the positive response accumulating NADH was then measured. The results showed that within a certain range, activation of PGK1 by 7979989 (10 μM-1 nM), Z112553128 (10 μM-1 nM), and AK-693/21087020 (1 μM-1 nM) caused a positive PGK response, thus promoting a positive GAPDH response to produce NADH. However, AK-693/21087020 inhibited PGK1 activity at 10 μM, and this difference may be due to the difference in drug-PGK1 interaction forces. The binding modes of these three compounds are shown in Figure 3. Theoretically, 7979989 and Z112553128 have the same molecular backbone, so they produce similar forces when they bind to the surrounding amino acid residues, and thus they are similar in terms of activity. While the structure of AK-693/21087020 is quite different from others resulting in different interactions with protein and different activities.

### 2.4 OGD/R Injury in PC12 Cells

**FIGURE 4:**
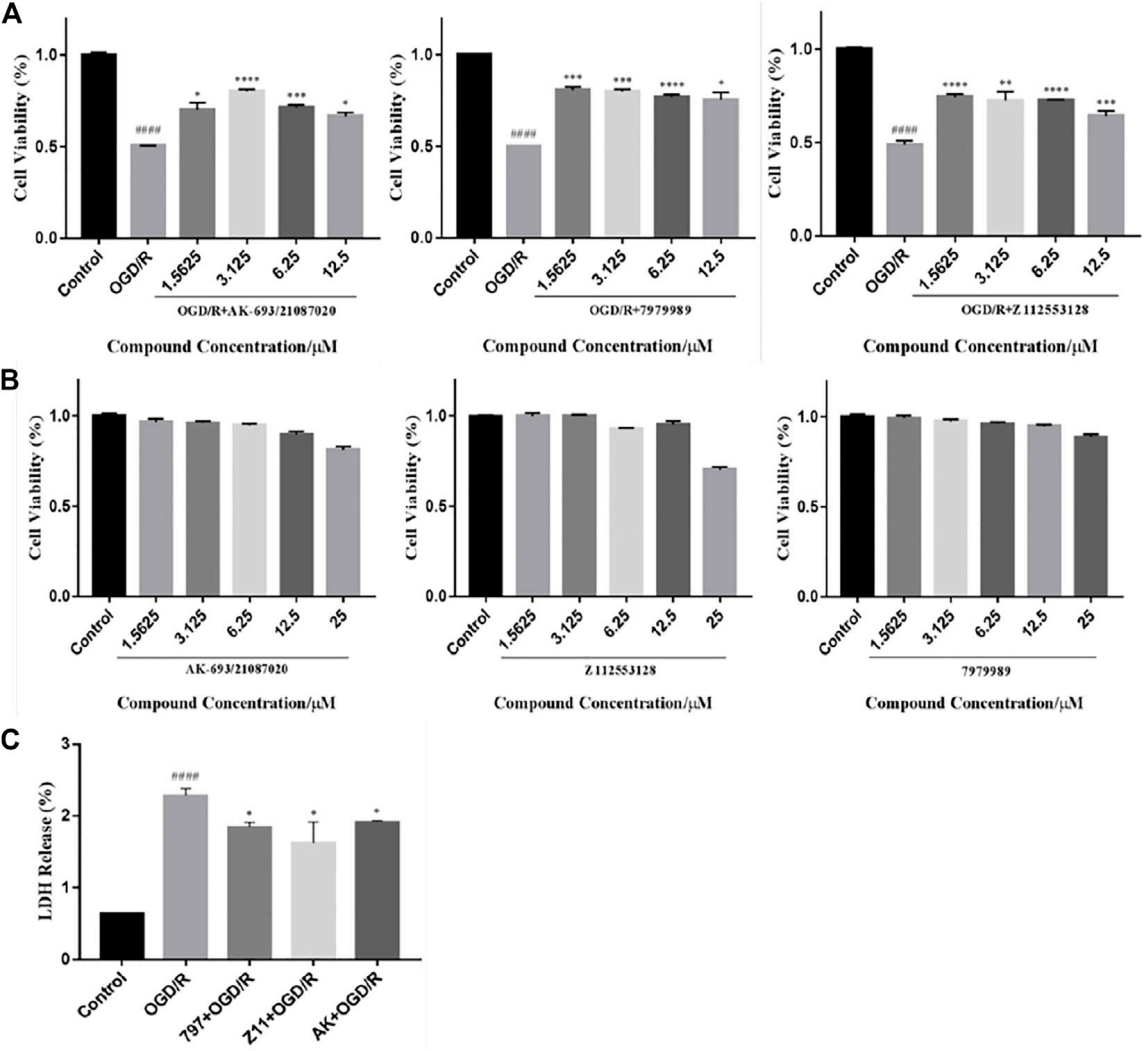
Compounds inhibit OGD/R-induced cell viability loss and cell apoptosis. **(A)** MTT assay showed that the candidate compounds (12.5–1.5625 μM) were safe for PC12 cells. Values obtained from three independent experiments are expressed as mean ± SD (% of control). **(B)** Protective potential of the candidate compound (12.5–1.5625 μM) on cell viability of PC12 cells exposed to OGD/R. **(C)** LDH release after OGD/R injury. Each bar represents the mean ± standard deviation (SD) calculated from three independent experiments. ####*p* < 0.0001 vs. control group; ****p < 0.0001; ***p < 0.005, **p < 0.01, *p < 0.05 vs. OGD/R group.

The biosafe concentrations of the compounds were determined by MTT assays. Cells were exposed to concentrations of candidate compounds ranging from 25 to 1.5625 μM for 24 h (Figure 4B). MTT assays showed that almost 100% of PC12 cells treated with 12.5 μM or lower concentrations survived compared to the controls, indicating that the different concentrations of compounds ranging from 12.5 to 1.5625 μM did not cause any significant decrease in cell decrease in viability. However, the higher concentrations reduced the cell viability (AK: 80.74 ± 3.66% of control value, *p* < 0.005 vs. control; 797: 89.61 ± 1.91% of control value, *p* < 0.01 vs. control; Z11: 70.30 ± 1.61% of control value, *p* < 0.0001 vs. control), suggesting that concentrations above 25 μM are detrimental to neuronal cells.

To explore the protective effect of candidate compounds against neuronal OGD/R injury, we treated OGD/R-injured PC12 cells with these compounds (12.5–1.5625 µM). The obtained data showed a significant decrease in cell viability after OGD/R injury (AK: 54.36 ± 5.76% of control value; 797: 53.74 ± 5.76% of control value; Z11: 52.39 ± 5.76% of control value, *p* < 0.0001 vs. control). Significant improvements in cell viability were observed in the compound groups at concentrations of 12.5, 6.25, 3.125, and 1.5625 μM compared to the OGD/R group (AK: 65.53 ± 3.15%, 71.53 ± 1.49%, 80.37 ± 1.19%, and 71.69 ± 4.32% of control value, respectively; 797: 72.37 ± 5.60%, 77.19 ± 1.02%, 78.45 ± 2.55%, and 80.86 ± 1.60% of control value, respectively; Z11: 65.65 ± 3.07%, 74.24 ± 0.38%, 74.78 ± 5.13%, and 76.57 ± 1.53% of control value, respectively) (Figure 4A). The graph does not show dose-dependence probably because hypoxic conditions increase the expression of hypoxia-inducible factor-1 (HIF-1). Under hypoxic conditions, most eukaryotic cells can convert mitochondrial respiration to increased glycolysis to maintain ATP levels. The presence of HIF-1 leads to increased expression of glycolytic enzymes such as PGK1, which accelerates the glycolytic process and correspondingly increases the level of lactate, a glycolytic product that leads to a decrease in intracellular pH, resulting in cellular acidosis and ultimately apoptosis. Although the compound activates PGK1 with neuroprotective effects, the elevation of PGK1 levels under hypoxic conditions causes cellular acidosis and eventually apoptosis.

LDH release was significantly increased in OGD/R-treated PC12 cells (228 ± 1.26%) compared to the control group (Figure 4C). A decrease in LDH release could be recorded following compounds treatment. OGD/R + AK (3.125 μM), OGD/R +797 (1.5625 μM), and OGD/R + Z11 (1.5625 μM) treatment reduced the values to 191 ± 0.45%, 184 ± 1.39%, and 162 ± 6.15%, respectively.

### 2.5 Compounds Reduce OGD/R-Induced Apoptosis

**FIGURE 5:**
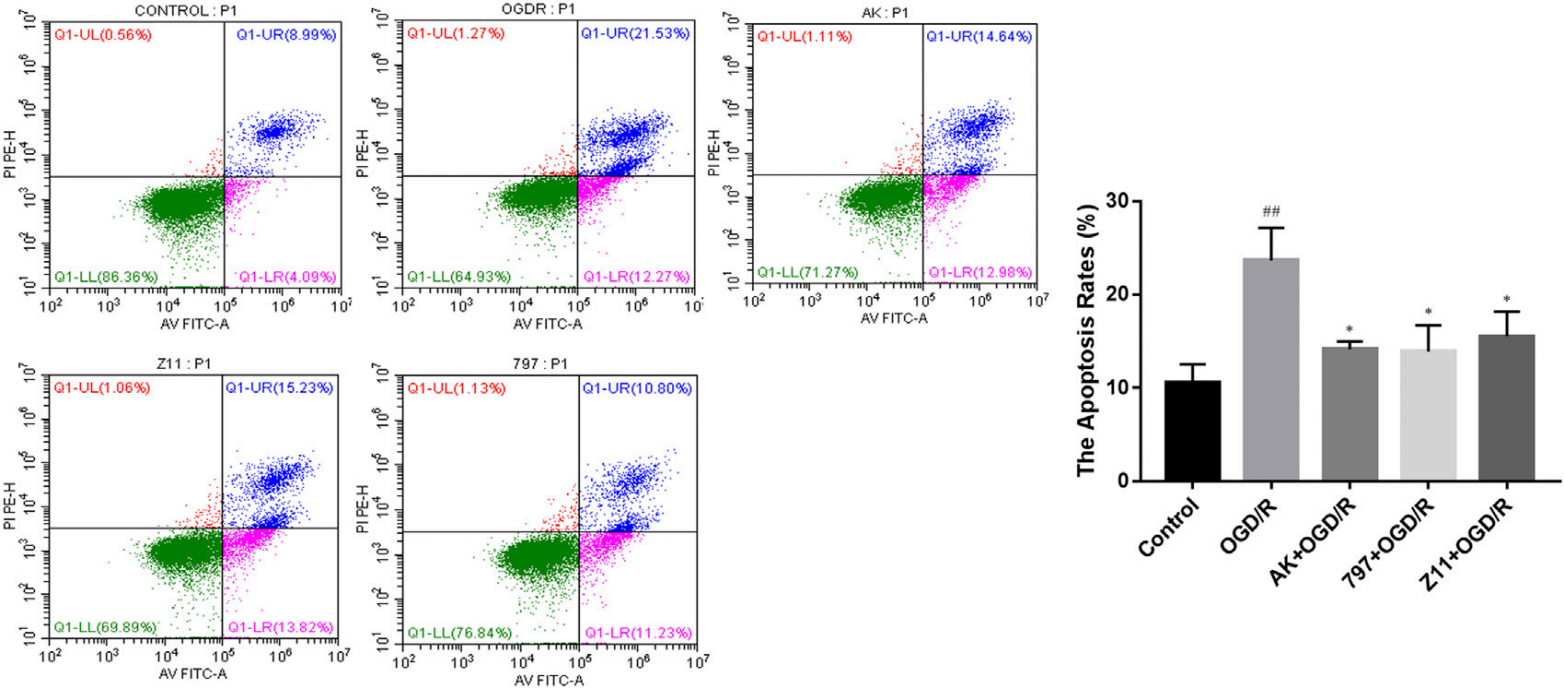
Compounds inhibit OGD/R-induced cell apoptosis. Flow cytometry images show that OGD/R-induced apoptosis in PC12 cells was rescued by the candidate compound (AK: 3.125 μM; 797: 1.5625 μM; Z11: 1.5625 μM). Each bar represents the mean ± standard deviation (SD) calculated from three independent experiments. ##*p* < 0.01 vs. control group; *p < 0.05 vs. OGD/R group.

The effect of compounds on OGD/R-induced apoptosis in PC12 cell was shown by flow cytometry using Annexin V-FITC/PI double staining. As shown in Figure 5, the apoptosis rate after OGD/R injury was much higher than the control group, while in the OGD/R + AK (3.125 μM), OGD/R +797 (1.5625 μM), and OGD/R + Z11 (1.5625 μM) groups, the apoptosis rate after OGD/R injury was significantly reduced, indicating that the compounds were effective in preventing OGD/R-induced apoptosis.

### 2.6 Cell Cytotoxicity Measurement

**FIGURE 6:**
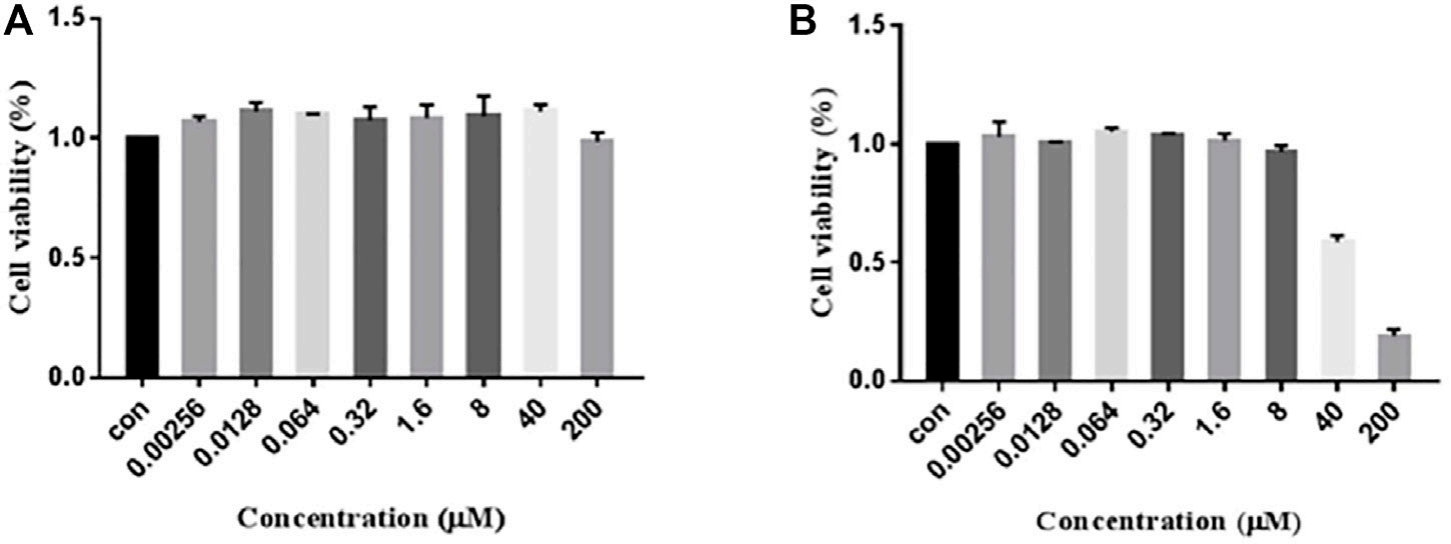
Cytotoxicity of 7979989 and Z112553128 on RAW264.7 Cells. RAW264.7 cells were treated with 7979989 and Z112553128 for 24 h, and cell viability was determined by the MTT assay. Each bar represents the mean ± standard deviation (SD) calculated from three independent experiments.

The MTT experiment was performed to ascertain whether 7979989 and Z112553128 have cytotoxicity on RAW264.7 cells. The results were shown in Figure 6, from where we can see that compound 7979989, from 0.00256 to 200 μM for 24 h, did not decrease the cell viability compared with the control group. As for Z112553128, the results appeared a decreased trend from the concentration 40–200 μM. Hence, in the following steps, we chose a concentration range of 0.0128–8 μM to verify preliminarily the anti-inflammatory properties by using the Griess method.

### 2.7 NO in LPS-Induced RAW264.7 Cells

Lipopolysaccharide (LPS) is a major component of the outer cell membrane of Gram-negative bacteria and is intrinsically important for inflammation caused by pathogens. When bound with LPS, the receptor TLR4 is initiated, thereby promoting downstream events. NF-κB drives a classical signaling pathway and gene regulation that is associated with various of diseases including inflammatory diseases. Activation of NF-κB initiates transcription and expression of pro-inflammatory genes, which then leads to excessive accumulation of various mediators such as tumor necrosis factor (TNF)-α, interleukin (IL)-β and IL-6. In this experiment, we finally determined the LPS (1 μg/ml) according to the conditions, and dissolved it in PBS. But for stimulation, the environment has no special requirements, just add LPS directly to the cell culture medium. Finally, by verifying the NO release of the LPS group and the non-adding group to determine whether the inflammation model is successful.

**FIGURE 7:**
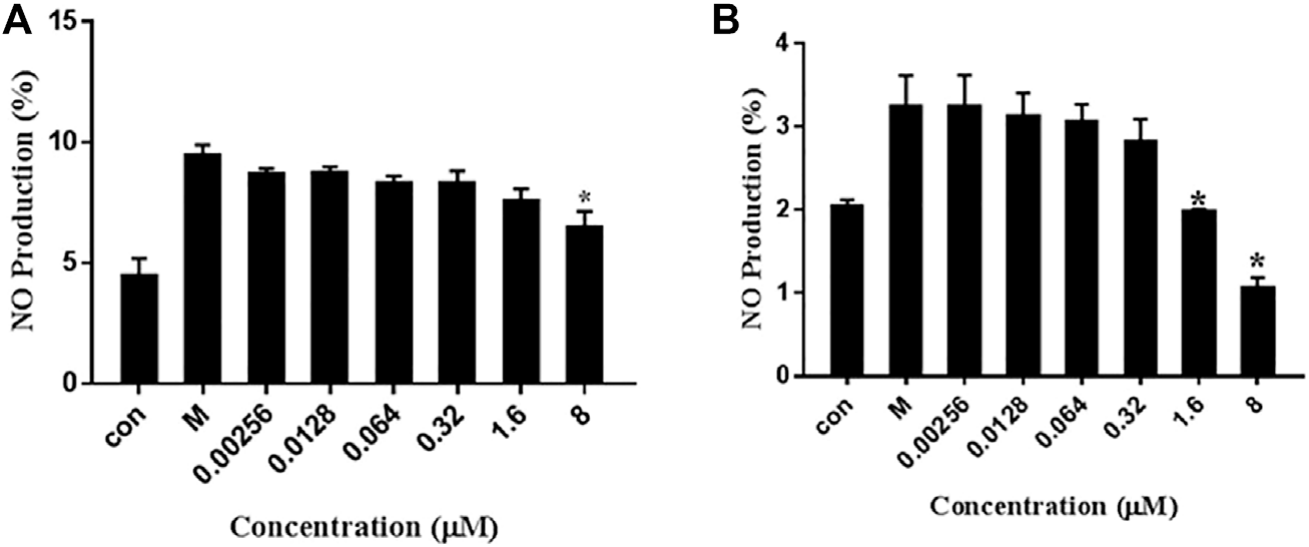
Effects of 7979989 and Z112553128 on the Production of NO in LPS-Induced RAW264.7 Cells. Inhibition of the **(A)** 7979989 **(B)** Z112553128 on LPS-induced nitric oxide (NO) in RAW 264.7 macrophages. Each bar represents the mean ± standard deviation (SD) calculated from three independent experiments. *p < 0.05 compared with M group (only LPS-treated group).

The RAW264.7 cells induced with LPS (1 μg/ml) were exposed to various concentrations of 7979989 and Z112553128 ranging from 0.00256 to 8 μM. The obtained results showed that NO concentration released by RAW264.7 cells increased significantly when added with LPS compared with control group without LPS, and the treatment with 7979989 and Z112553128 decreased the LPS-induced NO production. As shown in Figure 7, the two compounds have similar inhibitory trend on NO, and both illustrated significant inhibitory effects at the con-centration of 8 μmol/L. Then the suppression effect decreased with the decrease of compound concentrations, and disappeared at around 0.0128 μM. At the concentration of 8, 1.6 and 0.32 μM, they showed better inhibitory effect than other concentrations. Therefore, we chose 8, 1.6 and 0.32 Μm for subsequent experiments.

### 2.8 TNF-α, and IL-1β Induced by LPS

**FIGURE 8:**
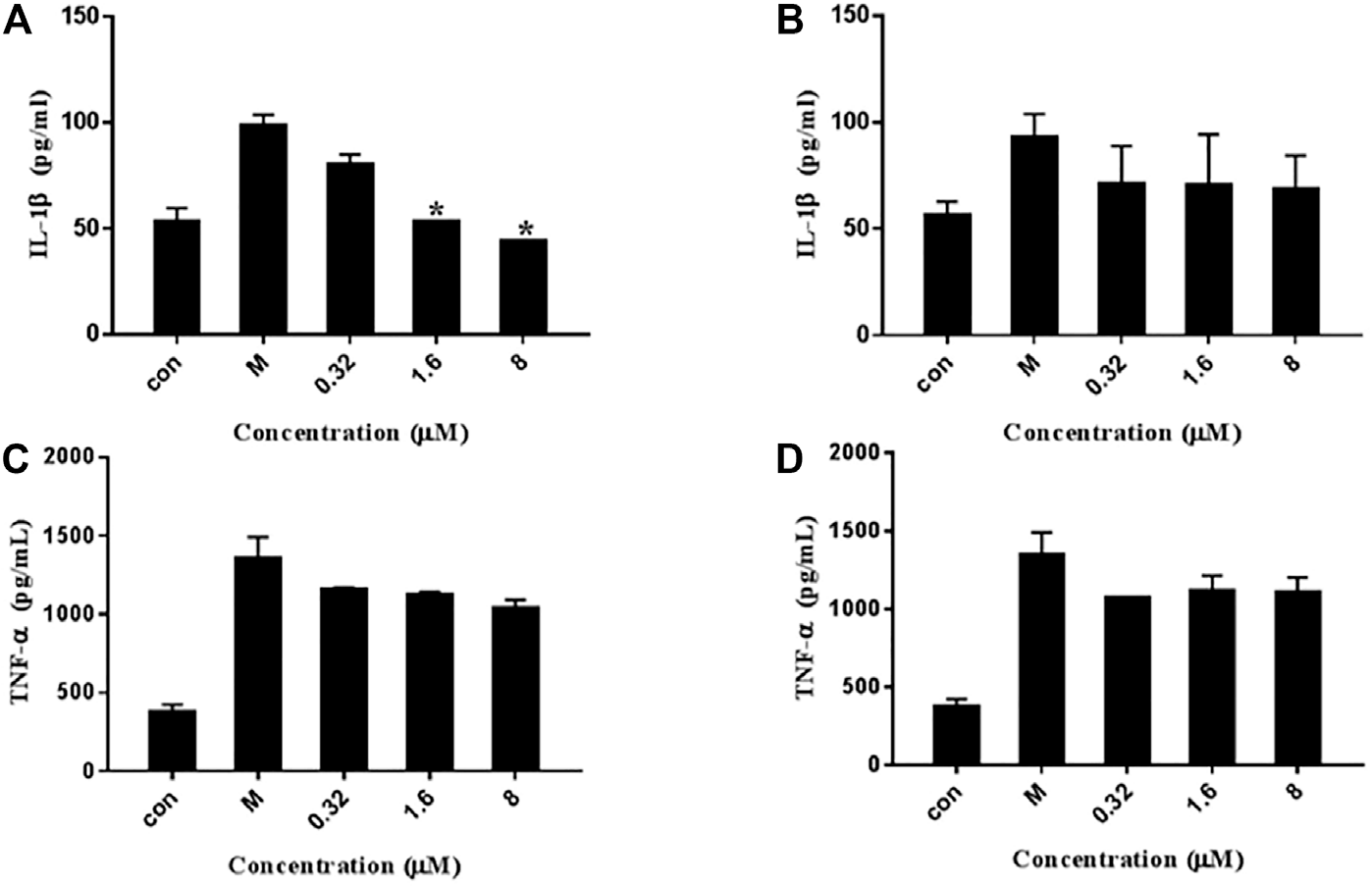
Effects of 7979989 and Z112553128 on TNF-α and IL-1β induced by LPS. In addition to an untreated control sample, RAW264.7 cells were pretreated with different concentrations of 7979989 and Z112553128 (8, 1.6, and 0.32 µM) for 1.5 h and then treated with 1 µg/ml LPS at 37°C for 24 h the culture supernatants of RAW264.7 macrophages were then detected by ELISA. **(A)** The levels of IL-1β inhibited by 7979989, **(B)** The levels of IL-1β inhibited by Z112553128, **(C)** TNF-α suppressed by 7979989, **(D)** The levels of TNF-α inhibited by Z112553128. Each bar represents the mean ± standard deviation (SD) calculated from three independent experiments. *p < 0.05 compared with M group (only LPS-treated group).

To investigate the inhibitory effect of 7979989 and Z112553128 on IL-1β and TNF-α production, we added the compounds to the RAW264.7 cells pre-treated with LPS for 1.5 h, and collected the cell supernatant after 24 h for centrifugation to conduct ELISA detection of different factors. As shown in Figure 8, compared with the unstimulated cells of control group, the level of IL-1β and TNF-α were increased significantly in LPS-induced cells of the model group. After adding 7979989 and Z112553128, the release of inflammatory factors produced by macrophages were inhibited to varying degrees. Shown in Figure 8A, the compound 7979989 displays an effective inhibitory effect on IL-1β production while it only slightly hampered the production of TNF-α (Figure 8C). In addition, Z112553128 has a specific characteristic of inhibiting the release of IL-1β and TNF-α as shown in Figures 8B,D, which is consistent with the previous NO results.

### 2.9 LPS-Induced iNOS and COX-2 Expression

**FIGURE 9:**
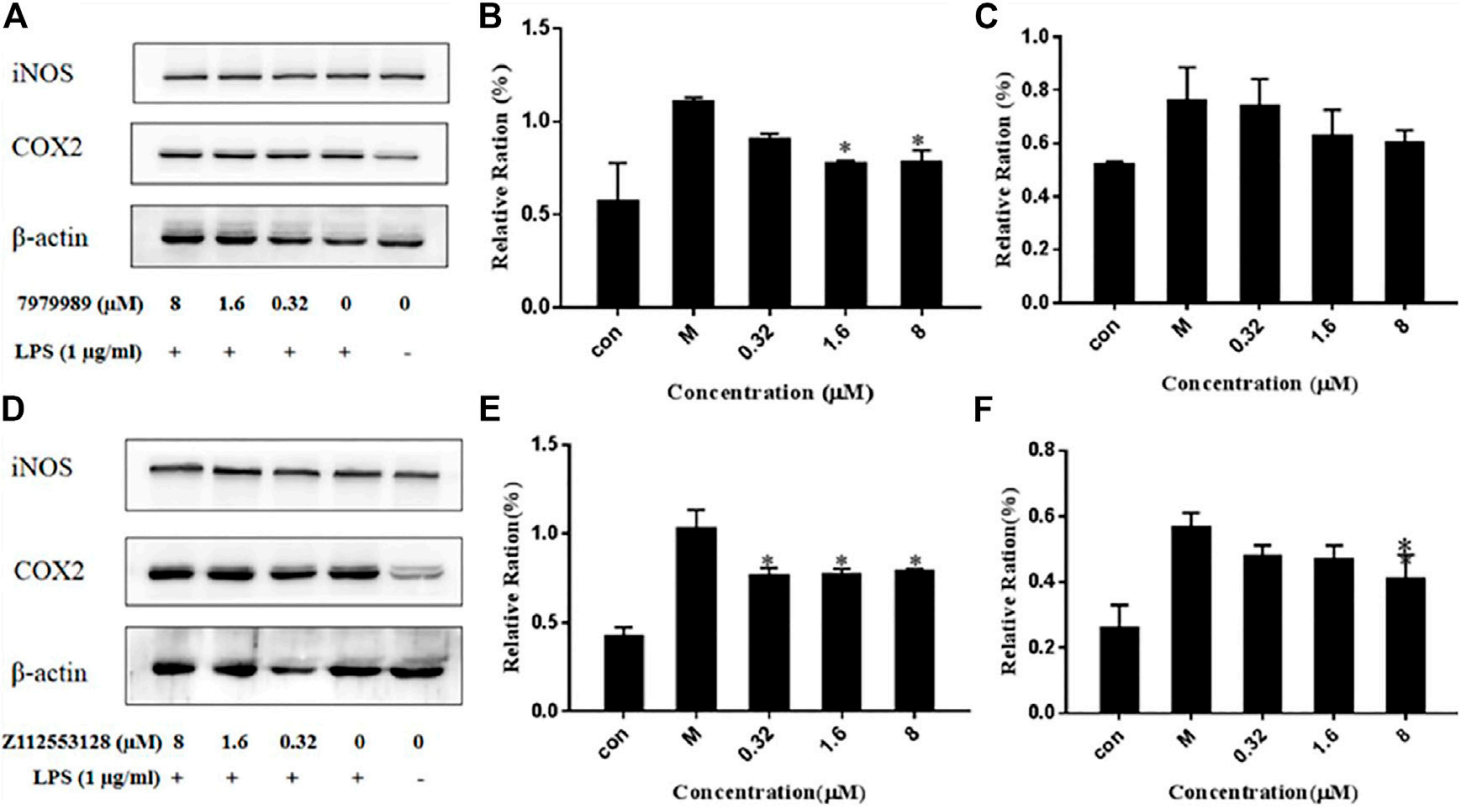
7979989 and Z112553128 decrease LPS-induced protein expressions of iNOS and COX-2. The protein expressions of iNOS and COX-2 were detected by western blot analysis. **(A)** Protein expression level after dealing with 7979989, **(B)** COX-2 protein expression level affected by 7979989, **(C)** iNOS protein expression level affected by 7979989, **(D)** Protein expression level after Z112553128 addition, **(E)** COX-2 protein expression level affected by Z112553128, **(F)** iNOS protein expression level affected by Z112553128. Each bar represents the mean ± standard deviation (SD) calculated from three independent experiments. *p < 0.05 compared with M group (only LPS-treated group).

To evaluate the effect of 7979989 and Z112553128 on pro-inflammatory mediators, we determined the expressions of iNOS and COX-2. Figure 9A shows that compound 7979989 could reduce the expressions of iNOS and COX-2 proteins (*p* < 0.05) compared with the LPS alone-treated model group and the appearance of iNOS and COX-2 were slightly weaker than LPS alone-treated model group. Figures 9B,C summarize the protein expression level and trend, where the levels of iNOS and COX-2 were significantly reduced. Compound Z112553128 also reduces the expressions of these two proteins in Figures 9D–F. Thus, both 7979989 and Z112553128 play essential roles in inhibiting the expression of inflammatory mediators such as iNOS and COX-2.

### 2.10 ROS Production Level Determination

**FIGURE 10:**
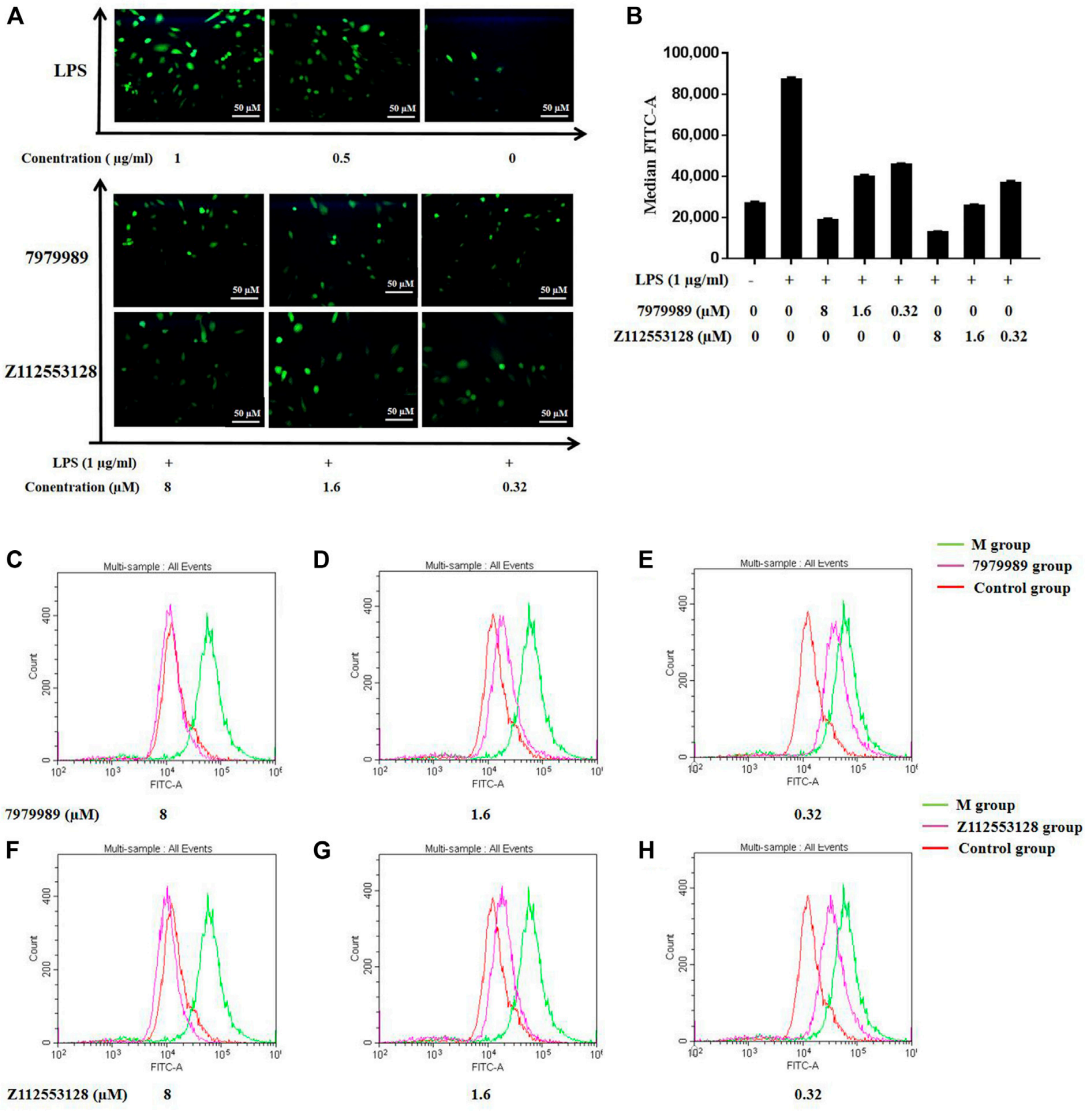
7979989 and Z112553128 inhibited LPS-induced intracellular ROS production. **(A)** Comparison of fluorescence staining images of ROS, **(B)** Relative fluorescence intensity analysis, Green represents the M group, red represents the control group, and pink represents the research group. Compared with the model group and the control group, the inhibitory effect of 7979989 and Z112553128 on the generation of ROS in cells is illustrated. **(C)** ROS production at 8 µM of 7979989, **(D)** ROS production at 1.6 µM of 7979989, **(E)** ROS production at 0.32 µM of 7979989, **(F)** ROS production at 8 µM of Z112553128, **(G)** ROS production at 1.6 µM of Z112553128, **(H)** ROS production at 0.32 µM of Z112553128. The results were presented in the above superposition of fluorescence spectra of all groups. *p < 0.05 compared with M group (only LPS-treated group).

To evaluate the effects of compounds 7979989 and Z112553128 on oxidative stress induced by LPS, fluorescence staining and flow cytometry were used to examine intracellular ROS production. Figure 10 shows that after LPS stimulation alone, the release of ROS was strongly stimulated as shown by the green normal distribution curve. After adding 7979989, the normal distribution curve had a significant shift in cytofluorogram to the left and almost reached a coincidence with the control group. Compared with the model group, the distribution of the Z112553128 also had an obvious shift to the left, indicating that it had an inhibitory effect on the production of reactive oxygen species in the cells. The inhibitory effect gradually weakened as the concentrations decreased, and a significant decline in the average fluorescence intensity was detected. Compared with the model group, the image also indicated a sharp decrease after treatment with 7979989 and Z112553128, while the average fluorescence intensity was significantly changed in the concentration range of 8 to 0.32 µM (*p* < 0.05). Therefore, compounds 7979989 and Z112553128 can reduce LPS-induced production of intracellular ROS.

## 3 Conclusion

**FIGURE 11:**
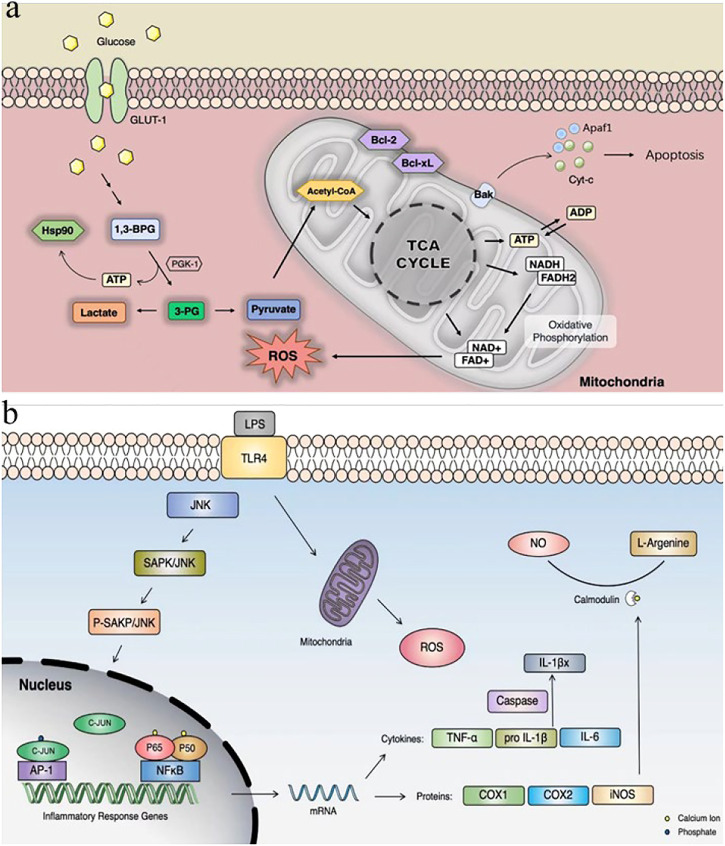
**(A)** PGK1 glycolysis and inhibition of apoptotic pathways; **(B)** LPS-induced cellular inflammatory pathways. GLUT-1: glucose transport; Cyt C: cytochrome c; Apaf-1: apoptotic protease-1.

Ischemic injury is a severe and complex pathological process involving peroxidative stress, neuroexcitotoxicity, and inflammatory activation. These chain reactions ultimately lead to apoptosis of neural cells (Jiang and Hao, 2020). The mechanism of apoptosis inhibition of the compounds found in this study may be similar to that of terazosin, by promoting the release of ATPase from PGK1 (Li et al., 2016), which then activates the activity of the molecular chaperone Hsp90, thereby resulting the enhance of its interaction with client proteins (Li and Buchner, 2013). Hsp90 can inhibit apoptosis through different mechanisms. The hallmarks of the apoptotic pathway are the involvement of mitochondria and the formation of apoptotic bodies. Cell death signals induce the release of cytochrome c (Cyt C) from mitochondria, which then binds to apoptotic protease-1 (Apaf-1), inducing the oligomerization and eventual recruitment of procaspase-9 to form apoptotic bodies (Figures 11A) (Cohen-Saidon et al., 2006). Hsp90 may interact with Apaf-1 to prevent the formation of apoptotic bodies (Pandey et al., 2000) or to prevent Bid cleavage (Zhao and Wang, 2004). Hsp90 can also inhibit apoptosis by stabilizing the phosphorylated Akt kinase (Sato et al., 2000).

This study is still only a small step towards the development of neuroprotective drugs. The etiology of stroke is complex and there are no models that can accurately predict the effects of treatment or pathogenesis. It is for this reason that we have used the *Drosophila* oxidative stress model, the neuronal cell oxygen glucose deprivation model, and the LPS-induced cellular inflammation model together to collectively verify its possible efficacy. It has long been shown that both enhanced PGK1 activity and increased glycolysis alleviate neurodegeneration (Cai et al., 2019), and that energy deficiency may be a pathogenic factor in the pathogenesis of neurodegenerative diseases, leading to neuronal dysfunction and subsequent death (Saxena, 2012). Our compounds were able to activate PGK1 activity, but whether they do so by increasing the ATPase activity of Hsp90 deserves further investigation.

Our study showed that 7979989, Z112553128 and AK-693/21087020 protected against oxidative stress and inhibited apoptosis in a model of hypoxia and glucose deprivation in *Drosophila*. Also 7979989 and Z112553128 inhibited LPS-induced cellular inflammation (Figures 11B). Our results suggest that these three compounds may modulate their effects by activating PGK1. Although the development of neuroprotective drugs is ongoing, the development of clinically effective neuroprotective agents remains elusive. Ongoing clinical trials of neuroprotection in stroke remind us of the future need for neuroprotective agents that synergise multiple cascade responses (Paul and Candelario-Jalil, 2021). There is great potential for these compounds to become new protective agents and this provides some material basis for our future research.

## 4 Materials and Methods

### 4.1 Computer-Aided Virtual Screening

#### 4.1.1 Protein Preparation

The 3D crystal structure of the target protein PGK1 (PDB ID: 3C39) was retrieved from the Protein Data Bank (PDB; https://www.rcsb.org/), with a resolution of 1.85 Å (Gondeau et al., 2008). The protein was prepared in Discovery Studio 2.5 (DS 2.5) (Accelrys Software Inc., San Diego, CA, United States) by removing all water molecules, adding hydrogen atoms, removing protein polymorphs, and supplementing with non-intact amino acid residues (Al-Balas et al., 2013).

#### 4.1.2 Ligand Preparation and Drug Likeness Screening

Ligands were downloaded from the Specs database with 400 natural products and the PubChem compound database with 72955 chemicals (1 February 2020) in 3D conformers. The ligands were prepared by rectifying their bond angles and bond orders and were subsequently minimized using CHARMm force field (Rampogu et al., 2019).

Firstly,the Lipinski’s rule of five (ROF) (Lipinski et al., 2001) and the ADMET (absorption, distribution, metabolism, excretion and toxicity) process (Zhou et al., 2016) were used to screen the drug-like properties. The Lipinski’s five rules are predicted by using the molecular properties in the computational small molecule protocol in DS 2.5, including molecular weight (MWT) ≤ 500, hydrogen bonded acceptor ≤10, hydrogen bonded donor ≤5, and ClogP ≤ 5 (or MLogP > 4.15). The ADMET descriptors in the molecular property calculation protocol were then used to predict the ADMET properties, where the solubility, absorption, and blood-brain barrier (BBB) thresholds were set as 3, 1, and 2, respectively. The screened ligands were subjected to docking analysis.

#### 4.1.3 Molecular Docking

The initial screening was performed in the Libdock module of DS 2.5. LibDock is a rigid docking method with an algorithm based on molecular dynamics annealing (Ren et al., 2020; Sharma et al., 2020). All molecules from the last step were docked into PGK1 active site, defined as 15 Å around the co-crystal ligand, with Libdock and individual LibDock scores were generated. Then all compounds were ranked accordingly to the LibDock scores, and the top ranked compounds were selected for the precise docking.

To further filter and obtain more accurate docking results, we used the Glide module of Schrodinger maestro 10.1 (Schrödinger, LLC, New York) in XP mode to perform accurate docking. The hit ligands from Libdock were converted to multiple conformations using the OPLS 2005 force field in Ligprep module (Han et al., 2020). The Protein Preparation Wizard (Prep Wizard) was used to prepare the proteins (Sastry et al., 2013). Calculations were run in XP mode (extra precision) using default settings, and the co-crystal ligand was used to define the center of the grid box. The parameters used for the docking score include lipophilic terms, metal interactions, coulomb interactions, Vander-Waals interactions, hydrogen bonding terms, and Pi-Pi interactions. The docked molecules were ranked according to the docking scores, and the top compounds with the highest docking score values were selected for the succeeding analysis.

#### 4.1.4 Similarity Searching

The canvas module (Canvas, Schrödinger, LLC, New York, NY) was utilized to classify the docking results based on similarity to obtain potential PGK1 activators with rich structural diversities. The similarity classification uses hierarchical clustering, and the cluster similarity threshold was set to 0.98, and the parameter fp-linear was used as a default value (Zhang and Muegge, 2006; Duan et al., 2010). Finally, compounds with suitable backbone and interactions were retained by considering receptor-ligand binding mode, ligand spatial site resistance, molecular backbone, hydrocarbon groups, N-heterocyclic compounds, amino and amide, halogen, carboxyl, and ester groups (Han et al., 2020).

### 4.2 Experimental Test and Verification

#### 4.2.1 PQ-Induced Oxidative Stress in *Drosophila*

We used the w^1118^ *D. melanogaster* strain to create an oxidation model with Paraquat dichloride (methyl viologen; PQ). *Drosophila* flies were reared in a biochemical incubator, where the temperature was set to 25°C and light exposure was set to 12-h/12-h LD cycles. *Drosophila* were raised on food containing 20 g of corn flour, 5.875 g of yeast, 1.625 g of agar powder, 34.375 g of sucrose, 0.5 g of sodium benzoate, and 3.1 ml of propionic acid dissolved in 1 L of water (Ueda et al., 2021). To investigate the role of compounds in the PQ-induced *Drosophila* model, PQ-treated (6 mg/ml, 24 h) w^1118^ was used. *Drosophila* were first treated with a 0.002% solution of the compounds configured in sucrose solution for 24 h and then treated with 6 mg/ml PQ. Then the effects on *Drosophila* were observed and studied. *Drosophila* without any treatment was used as reference controls. To make up compounds for treatment, the mother solution was first prepared with 5% sucrose solution in water (M/V) to 0.2% (M/V), and then diluted to 0.002% with 5% sucrose solution in water (M/V).

#### 4.2.2 Clonal Expression and Purification of hPGK1

To induce Pgk1 expression *in vitro*, the cDNA of human-derived Pgk1 was cloned into the pET28a (+) vector (Solarbio Science & Technology Co., Ltd., Beijing). Protein expression was induced in BL21 (DE3) chemically competent cells (Shanghai Angyu Biotechnology Co., Ltd.) with 0.5 mM IPTG. After lysis of cells in extraction buffer (20 mM sodium phosphate, 500 mM NaCl, 5 mM imidazole, 5% glycerol, protease inhibitor cocktail, pH 7.9), the supernatant was purified with nickel beads (QIAGEN, Germany). Elution buffer (20 mM sodium phosphate, 500 mM NaCl, 80 mM imidazole, protease inhibitor cocktail, pH 7.9) was used to elute hPgk1. The eluate was subjected to SDS-PAGE to identify the molecular mass and purity of the proteins (Chen et al., 2015).

#### 4.2.3 Activity Assay of PGK1

To measure the activity of PGK1, the purified recombinant human PGK1 protein (2 μg/ml) was mixed with the substrate (1.6 mM GAP, 1 mM β-NAD, 1 mM ADP, 20 ng/μl GAPDH) (Sigma Aldrich, St. Louis, MO, United States) in a buffer (20 mM Tris, 100 mM NaCl, 0.1 mM MgSO_4_, 10 mM Na_2_HPO_4_, 2 mM DTT at pH 8.6) for the reaction. Because the positive response of PGK can lead to a significant reduction in ADP levels, this would promote greater production of NADH by GAPDH. NADH can be detected at an absorbance of 339 nm. Therefore, the change in relative NADH levels from baseline was used to indicate the activity of PGK (Chen et al., 2015).

#### 4.2.4 Cell Culture and OGD/R Model

Rat pheochromocytoma cells (PC12 cells) purchased from ProCell (Hubei, China), were cultured in RPMI 1640 medium (Hyclone, GE Health Care Life Science, Little Chalfont, Buckinghamshire, United Kingdom) containing 10% fetal bovine serum (GIBCO, Australia), 1% penicillin and streptomycin in a humidified atmosphere of 95% air and 5% CO_2_ at 37°C. The medium was replaced every 48 h and cells grown at logarithmic phase with good growth and a density of 90% were passaged at a ratio of 1:3.

PC12 cells were inoculated in 96-well plates and cultured until apposed, and then were divided into control and OGD/R groups. The control groups were incubated with Dulbecco’s modified Eagle’s medium (DMEM, HyClone) in 95% O_2_/5% CO_2_ incubator. The OGD/R groups were added the corresponding concentration of the drugs and cultured in 95% O_2_/5% CO_2_ incubator for 24 h, and then changed to sugar-free medium containing the drug (Beijing Sunshine Biotechnology Co., Ltd.), which contained all standard components except glucose and incubate at 37°C for 12 h in a three-gas incubator (94% N_2_, 5% CO_2_, and 1% O_2_). The medium was replaced with DMEM medium containing glucose and incubated in a 95% O_2_/5% CO_2_ incubator at 37°C for 24 h for re-oxygenation.

#### 4.2.5 Lactate Dehydrogenase (LDH) Release Assay

The lactate dehydrogenase (LDH) release assay is a method to measure the membrane integrity. Structural disruption of the cell membrane due to apoptosis or necrosis results in the release of LDH from the cell plasma into the culture medium. A quantitative analysis of cytotoxicity can be achieved by measuring LDH activity. The assay was performed using a commercially available LDH assay kit for *in vitro* cytotoxicity assessment (NanJing JianCheng Bioengineering Institute). The absorbance at 450 nm was measured using an enzyme marker (TECAN, Infinite F50, Switzerland). The supernatant from each well of the culture plate was transferred to a fresh flat-bottomed 96-well culture plate and further processed for enzymatic analysis according to the manufacturer’s instructions (Agrawal et al., 2013).

#### 4.2.6 Detection of Apoptotic Ratio in PC12 Cells After OGD/R Treatment

To assess the effect of compounds on OGD/R-induced apoptosis, apoptotic ratio was assessed using the Annexin V-FITC/PI Apoptosis Detection Kit. The treated cells were trypsinized, harvested, washed three times with PBS, resuspended in 100 μl binding buffer at a concentration of 1–5*10^5^ cells and then incubated with a combination of 5 μl Annexin V-FITC and 10 μl PI staining solution for 15 min at room temperature in the dark. The fluorescence of FITC and PI was measured using a flow cytometer (BECKMAN COULTER) and quantified using CytExpert software. After the quadrants on the Annexin V-FITC/PI dot plot were positioned, live cells (Annexin V^−^/PI^−^), early/primary apoptotic cells (Annexin V^+^/PI^−^), late/secondary apoptotic cells (Annexin V^+^/PI^+^) and necrotic cells (Annexin V^−^/PI^+^) were distinguished (Liu et al., 2016).

#### 4.2.7 Cell Culture and Apoptosis Induction

The RAW264.7 macrophage line (Chinese Academy of Science) was maintained in DMEM supplemented with 13% fetal bovine serum (FBS) (Gemini, Gansu) and 1% penicillin streptomycin (P/S) at 37°C in a 5% CO_2_ humidified air environment. The cells were pre-incubated with or without the indicated concentrations of compounds for 2 h in serum free media, prior to the addition of LPS (1 µg/ml) (Sigma Aldrich, St. Louis, MO, United States).

#### 4.2.8 Measurement of NO

The RAW264.7 cells were seeded at 5,000 cells per well and cultured in 96 well plates. After incubation for 24 h at 37°C, the cells were treated with compounds at the indicated concentrations for 1.5 h in serum free medium, prior to the addition of LPS (1 µg/ml). Incubating again for another 24 h after adding the compounds, the supernatants were measured for NO production using the nitrate/nitrite assay kit (Nanjing Jiancheng Bioengineering Institute). NO was measured as the accumulation of nitrite and nitrate reductase, which were determined through Microplate reader using Griess reagent at an optical density of 540 nm.

#### 4.2.9 Determinations of IL-6, TNF-α, and IL-1β Levels

The RAW264.7 cells were seeded at a density of 2 × 10^5^ cells/well in a 24-well plate and cultured for 24 h at 37°C. The cells were treated with compounds at the indicated concentrations for 1.5 h in serum free medium, prior to the addition of LPS (1 µg/ml). After incubating again for 24 h after adding the compounds, the supernatants of the cell culture were collected. The concentrations of IL-6, TNF-α and IL-1β were measured using ELISA kits (Boster, Wuhan, China).

#### 4.2.10 Western Blot Analysis

After pre-incubation with or without compounds for 1.5 h prior to exposure to LPS (1 µg/ml), the RAW264.7 cells were harvested. The cells were washed with PBS and then blown from the bottom of culture dish. The cell pellets were obtained by centrifugation at 1,500 rpm for 6 min at 4°C. After adding the cell lysate, it was placed on the ice and shaken at a constant speed for 30 min, and then was centrifuged at 14000xg for 15 min at 4°C to obtain the whole cell lysates. The protein concentrations were determined by the Bradford protein assay (Bio Rad Laboratories, Inc.). The proteins were resolved using 10% SDS-PAGE. Then the isolated protein was electrotransferred to the polyvinylidene fluoride (PVDF) membrane. The non-specific site was sealed with Tris-buffered saline with 0.5% Tween 20 (TBST) containing 5% skimmed milk powder for 2 h. Proteins fixed to the membrane can react with specific antibodies. Subsequent visualization of antibody binding was carried out with Enhanced Chemi-Luminescence and the obtained images were analyzed by ImageJ software.

#### 4.2.11 Determination of Intracellular ROS Content

The treated RAW264.7 cells in the six-well plates were collected and washed with the serum-free medium, then incubated with the fluorescent probe 2′,7′-dichlorodihydrofluorescein diacetate (DCFH-DA, 10 µM) (Solarbio Science & Technology) at 37°C for 30 min. The cells were washed with PBS to remove the free DCFH-DA for 3 times and analyzed by flow cytometry. Fluorescein isothiocyanate-A (FITC-A) value was detected to describe the content of ROS. We observed the intensity of fluorescence under a fluorescence microscope.

### 4.3 Statistical Analysis

Results of experiment show the average value of the results from three independent repeated experiments. Statistical analysis was performed using the GraphPad Prism 5.0 statistical software. Data are expressed as means ± standard deviation (SD). Statistical analysis was carried out using one-way ANOVA followed by Tukey’s multiple comparison test, with *p* < 0.05 considered statistically significant.

## Data Availability

The original contributions presented in the study are included in the article/Supplementary Material, further inquiries can be directed to the corresponding authors.
